# A Review of Manufacturing Methods for Flexible Devices and Energy Storage Devices

**DOI:** 10.3390/bios13090896

**Published:** 2023-09-20

**Authors:** Yuntao Han, Yunwei Cui, Xuxian Liu, Yaqun Wang

**Affiliations:** College of Energy Storage Technology, Shandong University of Science and Technology, Qingdao 266590, China

**Keywords:** flexible electronics, flexible energy storage, flexible electronics manufacturing

## Abstract

Given the advancements in modern living standards and technological development, conventional smart devices have proven inadequate in meeting the demands for a high-quality lifestyle. Therefore, a revolution is necessary to overcome this impasse and facilitate the emergence of flexible electronics. Specifically, there is a growing focus on health detection, necessitating advanced flexible preparation technology for biosensor-based smart wearable devices. Nowadays, numerous flexible products are available on the market, such as electronic devices with flexible connections, bendable LED light arrays, and flexible radio frequency electronic tags for storing information. The manufacturing process of these devices is relatively straightforward, and their integration is uncomplicated. However, their functionality remains limited. Further research is necessary for the development of more intricate applications, such as intelligent wearables and energy storage systems. Taking smart wear as an example, it is worth noting that the current mainstream products on the market primarily consist of bracelet-type health testing equipment. They exhibit limited flexibility and can only be worn on the wrist for measurement purposes, which greatly limits their application diversity. Flexible energy storage and flexible display also face the same problem, so there is still a lot of room for development in the field of flexible electronics manufacturing. In this review, we provide a brief overview of the developmental history of flexible devices, systematically summarizing representative preparation methods and typical applications, identifying challenges, proposing solutions, and offering prospects for future development.

## 1. Introduction

**Figure 1:**
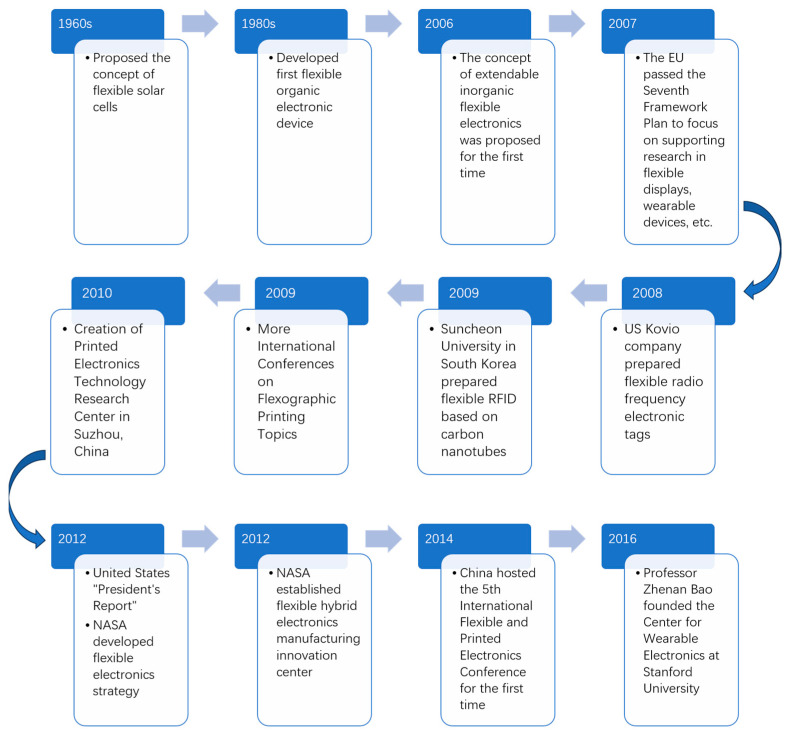
Development history of flexible electronics.

Although the concept of flexible solar cells was proposed in the 1960s and organic electronic devices with flexible characteristics were developed in the 1980s, it is only within the last two decades that international research on product flexibility has truly taken off. In 2006, the scientific community first introduced the concept of malleable inorganic flexible electronics [1]. In 2007, the European Union adopted the Seventh Framework Programme and invested billions of euros in research and development to focus on flexible displays, photovoltaics, sensing, and wearable devices [2]. In 2008, the United States Kovio Company achieved a significant milestone in the realm of printed electronics by successfully fabricating flexible radio frequency electronic tags (also known as radio-frequency identification: RFID) using inkjet-printed nanosilicon ink. In 2009, Soon Chun University in Korea advanced RFID technology further by employing roll-to-roll printing with inorganic nanomaterial inks like carbon nanotubes. Additionally, in the same year, international academic conferences on the theme of flexible printing were held in Europe and Asia, respectively [3,4]. Since 2009, there has been a turning point in international research and discussion focused on flexible applications, resulting in an increasing number of related associations and companies. In 2010, the Printed Electronics Technology Research Center was established by the Chinese Academy of Sciences Suzhou Institute of Nanotechnology and Nanobionics, and in 2014, China hosted the 5th International Conference on Flexible and Printed Electronics (ICFPE) for the first time [3,4]. In 2012, the U.S. Presidential Report identified flexible electronics manufacturing as a cutting-edge area for development; that same year, NASA formulated a strategy for flexible electronics and established the Flexible Hybrid Electronics Manufacturing Innovation Center in 2014 [2]. In 2016, Professor Zhenan Bao, a distinguished figure in the field of flexible electronics, established the Center for Wearable Electronics at Stanford University. Industry experts predict that the global flexible electronics market will experience a double-digit growth rate to reach $250 billion by 2025 [5]. The flexible display market is currently dominated by international companies such as Samsung and LG from South Korea and Toshiba and Sharp from Japan. Meanwhile, Flagship from Japan and M-FIEX from the United States have a significant advantage in the field of flexible circuits [6] (Figure 1).

**Figure 2:**
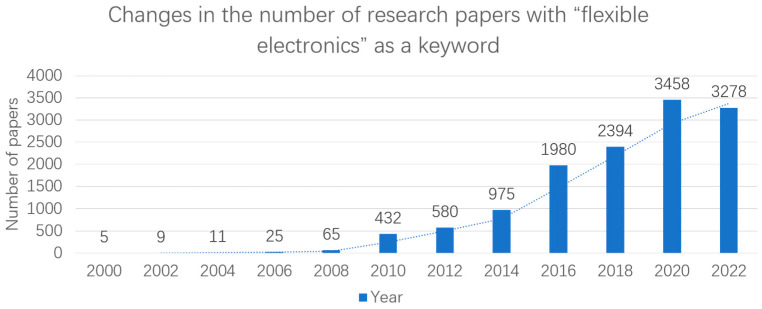
Changes in the number of research papers with the keyword “flexible electronics” in the past 20 years (data source: Web of Science).

Despite being a relatively new concept, flexible design has gained significant traction in both research and practical applications over the past two decades. As shown in Figure 2, since 2009, there has been an exponential increase in the number of papers containing keywords such as “flexible electronics” and “printing”. However, since 2020, the growth of papers in this field has shown a steady trend. The author believes that it may be that the research in the field of flexibility has become saturated, and this steady trend may continue in the future. On the other hand, there is a growing number of flexible application products that are capable of achieving full commercialization, such as flexible wiring [7,8] in various electronic devices and flexible radio frequency identification (RFID) tags [9,10,11]. More versatile products are expected to emerge in the future to better serve human needs. However, the development of more sophisticated and intricately flexible electronic devices is imperative.

There have been various methods for manufacturing flexible devices, including spin coating, scratch coating, spray coating, electrodeposition, and other simple techniques used to process flexible films. Additionally, etching, print manufacturing, and laser technology are employed to prepare circuit patterns on flexible substrates. There are also self-sustaining technologies available for the direct production of flexible films, including film extraction, electrostatic spinning, self-assembly, and other processes. These approaches have led to significant technological breakthroughs and the commercialization of some methods. However, it is important to acknowledge that there are still critical challenges that need to be addressed in the field of flexible device manufacturing [12,13,14,15,16,17]. These challenges include:

(1) Production cost: The cost of manufacturing flexible devices can be high, limiting their widespread adoption. Efforts should be made to develop cost-effective manufacturing processes.
(2) Functional expansion: While there have been advancements in the functionality of flexible devices, further expansion is needed to meet the diverse needs of different applications.
(3) Flexibility and stretchability: Improving the flexibility and stretchability of flexible products will enhance their durability and usability in various environments.
(4) Power consumption and detection accuracy: Flexible sensors should aim for lower power consumption without compromising detection accuracy to extend the battery life and enhance their performance.
(5) Biocompatibility: The biocompatibility of flexible substrates is crucial for their use in biomedical applications. Research should focus on developing biocompatible materials and ensuring their safety for use in contact with the human body.
(6) Application range: Expanding the application range of flexible devices will enable their utilization in various industries and sectors, leading to increased market demand.
(7) Capacity of flexible batteries: Enhancing the capacity and efficiency of flexible batteries will enable longer device operation and reduce the need for frequent recharging.
(8) Integration of multi-layer flexible electronic devices: Developing methods for seamlessly integrating multiple layers of flexible electronic devices will enhance their overall functionality and performance.

This review systematically categorizes diverse manufacturing methods and applications of flexible devices and highlights their respective advantages, disadvantages, and areas for improvement. By addressing the aforementioned challenges, the field of flexible device manufacturing can continue to progress and realize its full potential in various industries.

## 2. Simple Flexible Device Preparation

This section provides an introduction to simple methods for preparing flexible devices, including thin-film self-assembly, single-layer circuit design, and packaging for flexible energy storage. Self-supporting technology allows us to prepare thin film substrates that meet our desired requirements, and we also have the option to purchase commercial substrates. While complex flexible devices may require different preparation methods, it is important to note that the examples listed in this section are not limited to a single method. Combining multiple methods is necessary, depending on the specific requirements of the preparation process [18].

### 2.1. Self-Supporting Flexible Device Preparation

The use of commercially available flexible materials as substrates for flexible electronic devices is a time-efficient and convenient approach that supports commercial production. However, not all flexible materials meet the specific requirements of scientific research. Many flexible substrates, such as polyethylene terephthalate (PET) film and polyimide (PI) film, vary in thickness, flexibility, and surface treatment. Additionally, using the same material substrate from different manufacturers can lead to inconsistent experimental results. This is where self-supporting technology comes into play, allowing for the design and preparation of flexible materials to meet the desired outcomes.

The simplest method for preparing self-supporting films involves filtration, where precipitates are formed on filter paper and then converted into a flexible film through drying and other processes. Another common method is the spinning process, which converts polymer materials into fibers and integrates them into a flexible film. This method offers unparalleled flexibility and comfort when in contact with the human body. Self-assembly processes are also utilized, where the components in solution spontaneously aggregate to form gels that can be further processed into flexible films. These practical methods enable the design and manufacture of flexible materials with self-supporting technology.

#### 2.1.1. Filtration into Film

Suction filtration (also known as vacuum filtration), whose professional name is decompression filtration operation, is a method commonly used in the laboratory. It is particularly effective for separating solid particles from liquids, allowing for the preparation of flexible films by depositing them on filter paper. The assembly of a suction filtration setup typically includes a Buchner funnel, a suction filter bottle, a rubber hose, a suction pump, and filter paper. The pump is used to create a pressure difference between the inside and outside of the filter bottle, which results in the filtration process.

By using different pore size specifications of filter paper, it is possible to achieve the filtration of different-sized particles in solution. This enables the filtration of large particle impurities, leaving behind a solution containing only small particles. Once most of the liquid in the solution has been transferred to the extraction bottle through the air pressure difference, the desired substances are deposited on the filter paper. After removing the setup and the filter paper, the prepared flexible film can be obtained by undergoing a simple drying process. This method offers several advantages. It is relatively simple, with straightforward principles and experimental procedures. The experimental equipment for suction filtration is relatively low-cost, making it economically efficient. Additionally, except for the disposable filter paper, the other components of the setup can be washed and used multiple times, further reducing costs.

**Figure 3:**
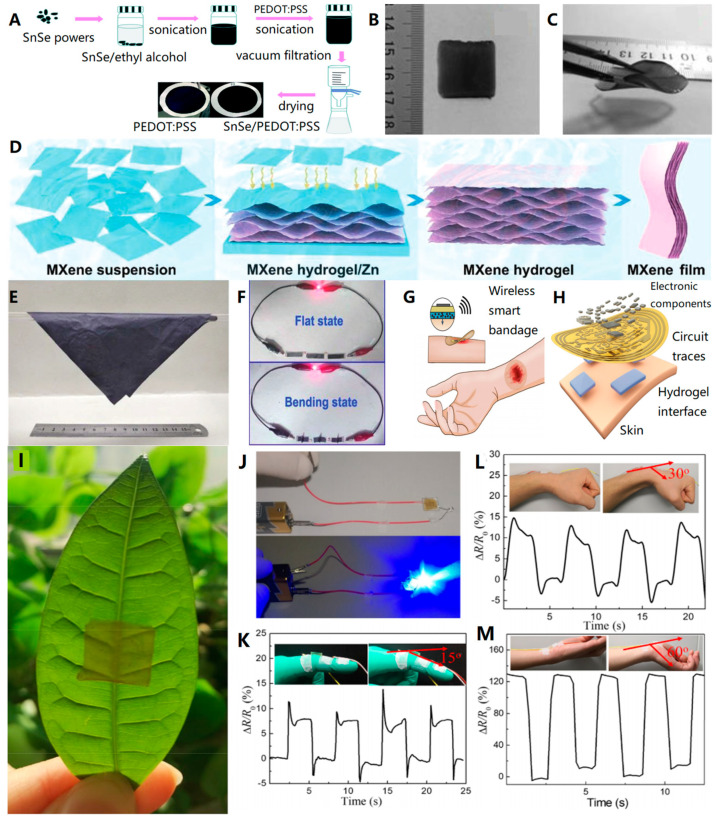
(**A**) Schematic illustration of the fabrication process for the flexible SnSe/PEDOT: PSS composite films [19]. Copyright 2020, RSC Publishing. (**B**,**C**) The prepared FeCo-PDMS composite film and its flexibility [29]. Copyright 2018, Elsevier. (**D**) Schematic diagram of the self-assembly process of MXene films. (**E**) Optical images of Mxene thin films show flexibility. (**F**) Optical images of LEDs lit in series by three devices in flat and bent states. It can be seen that the brightness of the lamp does not change when the power supply device is bent [30]. Copyright 2021, John Wiley and Sons. (**G**,**H**) Schematic and disassembled diagrams of the wireless smart bandage, including the FPCB and conductive adhesive hydrogel in contact with tissue (FPCB thickness ~100 μm) [33]. Copyright 2022, Springer Nature. (**I**) Representative image of a transparent hydrogel covering a leaf. (**J**) Photographs of circuits prepared based on hydrogels show that hydrogels have excellent electrical conductivity. (**K**–**M**) A conductive hydrogel sensor detects joint motion at different bending angles ((**K**): 15°, (**L**): 30°, (**M**): 60°) [34]. Copyright 2021, Springer Nature.

Yan et al. [19] used vacuum filtration to prepare flexible thermoelectric tin selenide (SnSe)/poly (3,4-ethylenedioxythiophene): poly (styrenesulfonate) (PEDOT: PSS) composite films (Figure 3A). These films showed great potential for use in wearable and flexible electronics. Similarly, MXene materials [20,21], which have gained popularity in recent years, can also be easily prepared into films using filtration [22]. During suction filtration, the thickness of the film can be controlled by adjusting the volume of the solution or the content of the sediment to be deposited [23]. Typically, to meet the requirements of self-support, the film thickness obtained through suction filtration ranges from microns to millimeters, depending on the mechanical properties of the material.

However, there are limitations to this method. Poor solution preparation, improper selection of filter paper specifications, and irregular operation can lead to unfavorable conditions during the filtration process. These include the solution passing through the filter paper without forming a film, the formation of a rough and uneven film surface, cracking of the film after drying, or the difficulties in removing the film from the filter paper. To ensure successful film preparation in future filtration experiments, it is important to focus on reasonable solution preparation, improvement of filtration equipment, and selection of high-quality and suitable filter paper. These factors play a crucial role in achieving the desired film properties and avoiding potential issues during the filtration process.

#### 2.1.2. Self-Assembly

Self-assembly [24,25,26,27] is a fascinating process that allows for the creation of ordered structures at a larger scale by organizing basic structural units through non-covalent bonding interactions. This process is spontaneous and does not require special devices, often utilizing water as the solvent. Self-assembly offers the advantage of controlled deposition and the ability to create membranes with molecular-level structures. By employing self-assembly techniques with different solutions, membrane materials can be designed to have various functions, such as optical, electrical, and magnetic properties, while maintaining flexibility. This makes them highly suitable for applications in flexible electronic devices. Additionally, combining self-assembly with magnetic control, lasers, redox reactions, and other methods can lead to unexpected and promising results.

The widely popular graphene material has a good role in the preparation of self-assembly. Combining chemically modified graphene (CMG) with laser beams, Jin et al. [28] successfully induced directed self-assembly of block copolymer (BCP) films. This method can be applied to flexible or complex substrates and avoids radiation damage to organic materials. This technology opens up possibilities for the fabrication of flexible electronic devices with tailored morphologies. Yuan et al. [29] combined mixture deposition, spin coating, and magnetic field-induced self-assembly to fabricate flexible and anisotropic polydimethylsiloxane (PDMS) composite films filled with FeCo nanochains. These composite films exhibit higher tensile strength compared to conventional pure films (Figure 3B,C), making them suitable for applications requiring highly stretchable devices. The thickness of the test samples used in the experiments was 1.3 +/− 0.2 mm (the thickness of the polymer film can be controlled through spin coating speed and the polymer viscosity).

The flexibility and stability of MXene films prepared through self-assembly strategies have been demonstrated by Zhao et al. [30]. These films are created through simultaneous reduction and self-assembly methods (Figure 3D,E), resulting in inflexible materials with a porous structure. The films exhibit excellent tensile strength and Young’s modulus, indicating their mechanical stability (Figure 3F). The thickness of the MXene films is 70.5 μm. Moreover, the MXene film maintains a constant resistance even when subjected to different bending states, highlighting its excellent conductivity. The researchers were able to utilize the Mxene films to create flexible supercapacitors with stable electrochemical properties, even in the bending state.

Self-assembly technology has enabled the development of flexible materials that can be seamlessly integrated with other electronic devices. In the medical field, these materials have been applied to the surface of human skin for medical treatment [31,32]. Jiang et al. [33] prepared a conductive adhesive hydrogel based on PEDOT: PSS material with low contact impedance, high toughness, and tunable adhesion by solution self-assembly technique. By combining this hydrogel with a flexible printed circuit board (FPCB), they designed a “smart” bandage that can perform real-time physiological detection and proactive intervention for chronic wound healing (Figure 3G,H). After simple treatment, such as gentle heating, the gel can be easily removed from the skin, reducing secondary damage to wound tissue.

Jing et al. [34] developed conductive transparent dipeptide hydrogels using self-assembly technology. These hydrogels had a thickness of 2 mm and exhibited good biocompatibility and multifunctionality. They also had similar physicochemical properties to human tissue, making them suitable for use in wearable biosensors (Figure 3I). The researchers created a biosensor by attaching hydrogel to the human body, as shown in Figure 3K–M. When the joints move, the biosensor detects regular resistance changes. This indicates that biosensors can accurately measure the tiny movements of the human body. This technology has great potential for applications in the field of biosensors.

Self-assembly technology offers the ability to produce films with varying thicknesses, ranging from microns to millimeters, to meet diverse requirements. This control over film thickness is achieved by manipulating the parameters of the self-assembly solution and refining operating procedures. Additionally, the mechanical properties of the material itself significantly influence film thickness. The simplicity and device-independent nature of self-assembly technology have made it widely used in the fabrication of flexible films.

Looking ahead, there are several areas where further advancements can be made. Firstly, optimizing the performance of self-assembled raw materials is crucial. This involves exploring ways to enhance the properties and functionalities of these materials, ultimately improving the quality and performance of the resulting films. Secondly, there is immense potential in skillfully combining self-assembly technology with other preparation processes, such as laser-induced self-assembly, magnetic field-induced self-assembly, and synchronous reduction self-assembly. Such integration can unlock new possibilities and lead to more sophisticated and tailored fabrication techniques. Lastly, the design of self-assembly products should prioritize compatibility with other flexible products and applications. By ensuring seamless integration with existing flexible products, the potential for innovative and versatile applications can be maximized.

#### 2.1.3. Spinning

Spinning, also known as chemical fiber forming, involves the conversion of certain polymer compounds into a solution or melt, which is extruded through spinneret pores to form chemical fibers. Electrostatic spinning [35,36,37,38] is a unique form of electrostatics that breaks down polymer fluids into tiny jets that can travel long distances and solidify into nanometer-diameter filaments. This approach allows the production of polymer filaments of nanometer diameter. This method enables the production of polymer fibers with desirable properties, such as processing flexibility, wearing comfort, excellent durability and maintainability, cost-effectiveness, and scalability. As a result, flexible devices based on fiber materials offer significant advantages and potential over devices made from other materials [39,40]. Electrostatic spinning has emerged as a prominent method for effectively preparing nanofiber materials due to its simple manufacturing device, low spinning cost, wide range of spinnable materials, and controllable process.

**Figure 4:**
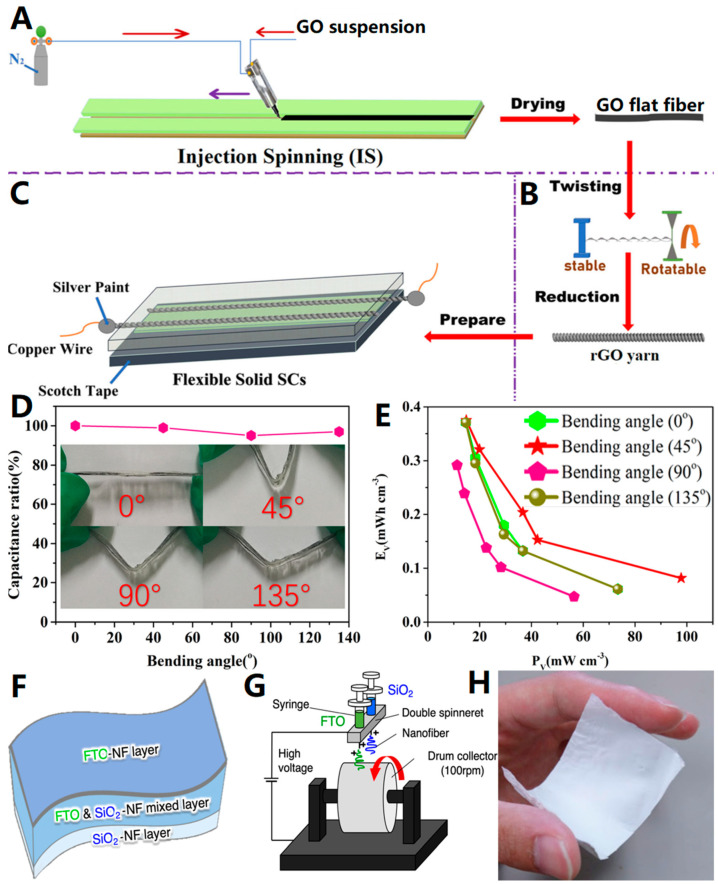
(**A**–**C**) Schematic illustration of the preparation process of rGO yarns and SCs. Where (**A**) is a diagram of the IS for preparing GO flat fibers. (**B**) Twisted and reduced. (**C**) Instructions for Flexible Solid SCs (GFSCs) preparation. (**D**) Capacitance change of SC under different bending angles (0°, 45°, 90°, 135°), and (**E**) Relationship between energy density and power density. It could be seen that the SC has an energy density as high as 0.37 mWh cm^−3^ and a power density of 14.7 mW cm^−3^ when the bending angle is 45° [41]. Copyright 2020, Springer Nature. (**F**) To overcome the brittleness of FTO-NF fabrics, a three-layer structure of FTO-SiO_2_ composite NF nonwovens was prepared. The bottom was a support layer composed of amorphous SiO_2_ NFs; the intermediate layer composed of FTO-SiO_2_ composite NFs was mainly responsible for electrical conduction; the top was the electrical contact layer; and the surface electrodes were only made of FTO-NFs. (**G**) Arrangement of a double spinneret and a rotating drum collector. (**H**) Photo of a prepared FTO-SiO_2_ composite NF fabric (thickness is about 220 μm) [42]. Copyright 2021, Springer Nature.

The spinning process enables the processing of graphene-based materials and exhibits excellent properties. Ma et al. [41] used injection spinning (IS) to directly spin flat fibrous graphene oxide (GO) (thickness range 5–15 μm) and produced reduced graphene oxide (rGO) yarns by further processing (Figure 4A–C). The resulting flexible solid supercapacitors (SCs), based on this yarn, demonstrated remarkable flexibility, cycling performance, capacitive behavior, and stability. Moreover, the overall performance of SCs remained largely unchanged even after being stored in the air for 4 months. Figure 4D illustrates the specific capacitance of the assembled SCs at different bending angles, revealing that the capacitance is independent of the bending angle. This characteristic is advantageous for practical applications of e-textiles (Figure 4E shows the relationship between SC energy density and power density). The fabrication of GO flat fibers with optimal morphology at the microscopic level through this method presents a novel approach for producing graphene fibers suitable for diverse designs and applications of flexible materials, especially wearable electronic devices.

Yoshinaga et al. [42] demonstrated the fabrication of conductive nanofiber (NF) fabrics containing fluorine tin oxide (FTO) for flexible electronics applications by electrospinning (Figure 4F,G). These fabrics exhibited a minimum resistivity of 5.19 Ω·cm and could experience only a 4.9% increase in resistance after 100 bending cycles, highlighting their excellent electrical conductivity, flexibility, and stability. Figure 4H shows a composite NF fabric that remained intact even when handled, emphasizing the excellent performance of electrostatic spinning in producing flexible materials with superior electrical properties. Notably, Yoshinaga et al. also pointed out that in subsequent work, to better apply electrospun fabrics to flexible electronics, the raw material solution of electrospinning can be improved in a targeted manner to further reduce its resistivity.

**Figure 5:**
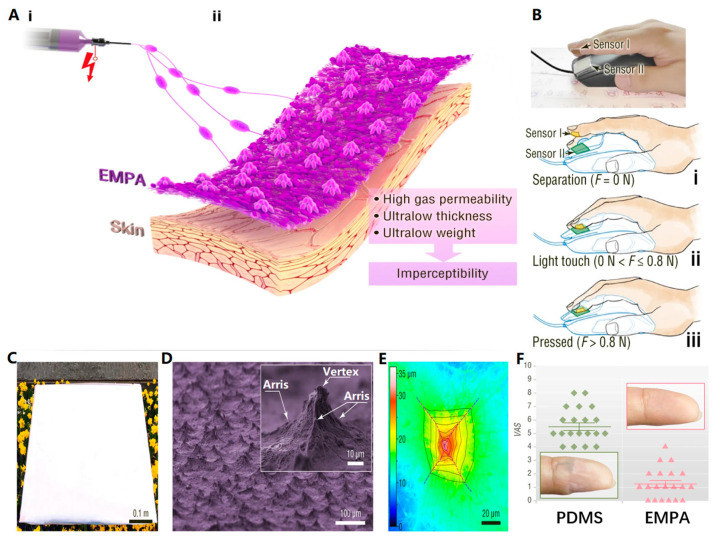
(**A**) Schematic illustration of (**i**) fabrication and (**ii**) structure. (**B**) The picture showed the finger operation monitoring of mouse click and three different states: (**i**) detached; (**ii**) tapped; and (**iii**) pressed. (**C**) Photographs based on large-area EMPA films. (**D**) SEM image of EMPA; the inset shows a magnified SEM image of an electrospun micropyramid. (**E**) Laser confocal microscope (LCM) image of electrospun micropyramids; the black dotted line and purple dotted line are the contour lines and alignment lines of the electrospun micropyramid structure, respectively. (**F**) When both devices were attached to the fingertips, participants reported any sensations, which were assessed on a visual analog score (VAS) (0–10, where 0: no sensory disturbance; 10: extreme discomfort). Horizontal bars represented mean values, and error bars were the standard error of the mean. The insets within the green and pink boxes showed the skin of the fingertip after 7 h of coverage with the different skin devices [43]. Copyright 2022, Springer Nature.

In 2022, Zhang et al. [43] successfully utilized electrospun self-assembly technology to create unique, ultrathin, ultralight, and permeable electrospun micropyramid arrays (EMPA). These arrays were used to develop various flexible electronic devices that can be seamlessly integrated into daily life and information detection (Figure 5). Specifically, the EMPA-based sensors are ideal for continuous health monitoring of specific individuals (e.g., drivers and e-sports players) without disrupting their normal activities (Figure 5B).

To evaluate the practicality of the EMPA device, multiple experimenters wore it on their fingertips. As seen in Figure 5F, the device had minimal impact on their fingers even after prolonged use, unlike the PDMS membrane-based device used for comparison. The experimenters reported that the PDMS device affected their work and daily lives, as evident from the wrinkling and whitening of the skin in Figure 5F. In contrast, the skin wearing the EMPA remained unaffected, demonstrating its excellent user experience. Furthermore, EMPA exhibits remarkable cooling capabilities, reducing skin temperature by 4 °C compared to direct sunlight exposure. The performance and application of EMPA can be further enhanced by optimizing its materials and structure. These findings highlight the vast opportunities for electrospun technology and EMPA in the design of future flexible wearable electronics that offer seamless human-computer interaction with minimal sensory interference.

In terms of flexible manufacturing, spinning technology offers several advantages over other processes. Firstly, spinning equipment is more cost-effective and easier to maintain compared with laser equipment and high-precision inkjet printers, resulting in significant time and effort savings. Secondly, due to their production process, spinning products closely resemble traditional clothing in texture and can be worn comfortably without causing sensitization issues, making them ideal for human health information monitoring. This capability sets spinning products apart from other flexible preparation processes. However, it is important to acknowledge the limitations of the spinning process. Since spinning primarily involves the production of sprayed nanofibers, the range of products that can be created through spinning is somewhat limited, mainly encompassing spun yarns and flexible films. Therefore, spinning is often used in conjunction with other methods to manufacture a wider range of flexible products. Nevertheless, considering that spinning products closely resemble traditional fabrics in texture, they are undeniably the optimal solution for manufacturing flexible smart wearable devices. Future research can focus on developing additional forms of spinning technology and flexible products to further advance the flexible market, particularly in the field of flexible smart wearables, in a more mature and professional direction.

Although the principles of suction filtration, self-assembly, and spinning differ significantly, they are all commonly used methods for preparing self-supporting flexible films. In terms of cost-effectiveness, suction filtration requires simple and inexpensive equipment, while self-assembly does not require any special equipment, making both methods economically advantageous. In contrast, spinning necessitates relatively expensive spinning equipment. Regarding precision control, spinning offers superior control over film thickness compared to suction filtration and self-assembly. However, all three methods can achieve the production of thin films ranging from microns to millimeters in thickness. Furthermore, spinning not only allows for the creation of films but also enables the production of various flexible products through yarn spinning.

### 2.2. Device Preparation Based on Flexible Substrates

In addition to utilizing self-assembly technology for the preparation of flexible substrates, it is crucial to focus on functional and product design. In many instances, opting for pre-made flexible substrates that meet the required specifications can enhance efficiency and save preparation time. Nowadays, the market offers a wide range of flexible substrates with different materials and properties, including popular options like PET film and PI film. These substrates can be customized according to specific parameters such as thickness, flexibility, and surface tension. The direct utilization of these flexible substrates can streamline the overall preparation process of flexible devices, especially when combined with other processes such as inkjet printing, electrochemical deposition, and laser technology. This clever integration enables high-volume automated production, enhancing efficiency and reducing costs, thereby facilitating commercial applications. For successful commercial flexible manufacturing, achieving high efficiency and low cost is of utmost importance.

#### 2.2.1. Scraping, Spin Coating, and Spray Plating

**Figure 6:**
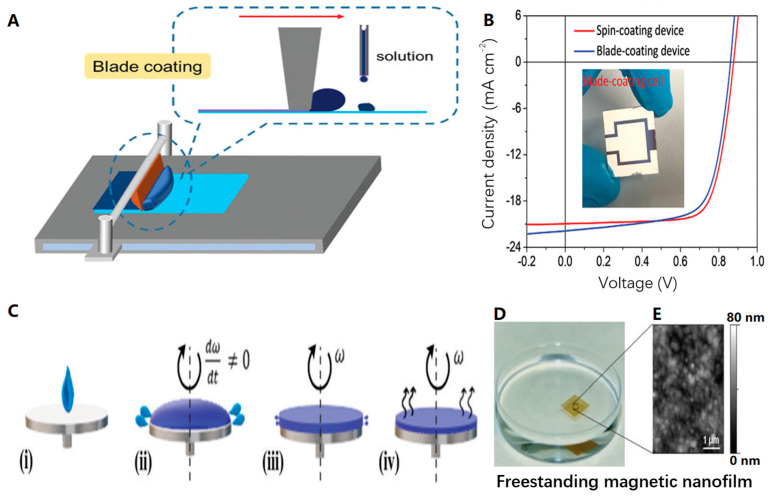
(**A**) Schematic diagram of the scraping process. (**B**) J–V curves of a 1 cm^2^ blade coating device; the inset is the actual device [49]. Copyright 2019, John Wiley and Sons. (**C**) Schematic diagram of spin-coating process stages: (**i**) dispensing stage; (**ii**) substrate acceleration stage; (**iii**) steady liquid outflow; and (**iv**) solvent evaporation. (**D**) The prepared nanofilm floats on the water’s surface. (**E**) Atomic force microscope scan of the nanofilm [50]. Copyright 2021, Royal Society of Chemistry.

In the field of flexible device manufacturing, there are several simple and straightforward methods for treating a flexible substrate, including scraping, spin coating [44,45,46,47,48], and spray coating. The scraping method, also known as blade coating, involves fixing the substrate onto a platform and dripping a solution onto one end of the substrate. As the scraper moves from one end to the other, the solution is transformed into a thin film. By controlling the size of the squeegee groove, we can regulate the thickness of the film. Figure 6A depicts the process of Cui et al. [49] using the scrape coating method when manufacturing solar cells. Additionally, Figure 6B displays the J–V curve of the 1 cm^2^ blade coating device, demonstrating that the solar cells fabricated using the blade coating method outperform those made using the traditional spin coating method. These illustrations provide an actual representation of the blade coating installation.

In the spin coating method, the substrate is placed on the suction cup of the spin coating machine and secured through vacuum adsorption. An appropriate amount of solution is then added dropwise to the center of the substrate. As the suction cup rotates, centrifugal force causes the solution to spread evenly, resulting in the formation of a thin film on the substrate. (Figure 6C illustrates the spin-coating process employed by Moreira et al. [50] in their design of an independent film for biomedicine; Figure 6D,E shows the prepared nanofilm and its microscope scan). The thickness of the film can be controlled by adjusting the rotational speed. It is worth noting that the edge effect during spin coating may lead to the formation of a thicker edge around the film, which requires attention to detail.

Spray plating is a technique that involves spraying a liquid or molten material onto a substrate’s surface using a power unit to create a complete cover layer. When combined with a mask stencil, it can be used to spray specific patterns onto the substrate surface with a certain level of accuracy. In traditional industrial design, plating the surface of a workpiece is a common practice to enhance its corrosion resistance, heat resistance, wear resistance, and thickness.

Scraping, spin coating, and spray coating processes are widely used in various fields because of their simplicity, ease of operation, and applicability in flexible device design. Many examples in this paper can be integrated into the overall process of flexible design. For instance, Wang et al. [51] employed spin coating, masking, and spray coating processes in their intricate design of skin electronic devices featuring stretchable transistors. Therefore, these methods have become indispensable in the manufacturing process of numerous flexible products.

#### 2.2.2. Deposition

Deposition is a material preparation technique commonly used in physical and chemical experiments. It involves the continuous deposition of solid particles suspended in a liquid. There are various forms of deposition, including physical vapor deposition [52], chemical vapor deposition [53,54,55,56,57], electrochemical deposition (electrodeposition) [58,59,60,61], biodeposition, and others.

Physical vapor deposition is a technique that involves the deposition of thin films by vaporizing a solid material and condensing it onto a substrate surface. Chemical vapor deposition, on the other hand, utilizes one or more gaseous or vaporous substances that undergo a chemical reaction at a certain temperature to produce a solid deposit on the substrate surface. Electrochemical deposition, or electrodeposition, involves the deposition of metal layers through a redox reaction on the substrate surface.

In the field of flexible electronic device design, particularly in the preparation of flexible thin films, two commonly used methods are electrodeposition and chemical vapor deposition. Among these, electrodeposition is widely preferred due to its simplicity in principle and operation. It is commonly employed in the preparation of electrodes and collectors for flexible energy storage. On the other hand, while the principle of chemical vapor deposition is theoretically simple, its realization process is demanding and requires expensive equipment. This makes it less cost-effective and unsuitable for volume production.

However, chemical vapor deposition also offers distinct advantages. As a relatively new technology for preparing inorganic materials, it allows for substance purification and ensures high product purity, thereby enhancing performance. This advantage cannot be achieved through traditional deposition methods like electrochemical deposition. Additionally, the vapor deposition method is well-suited for coating complex substrate surfaces, including granular materials and substrates with grooves, furrows, and holes. Therefore, when strict requirements for high purity of the prepared material are necessary, chemical vapor deposition is undoubtedly the optimal choice.

**Figure 7:**
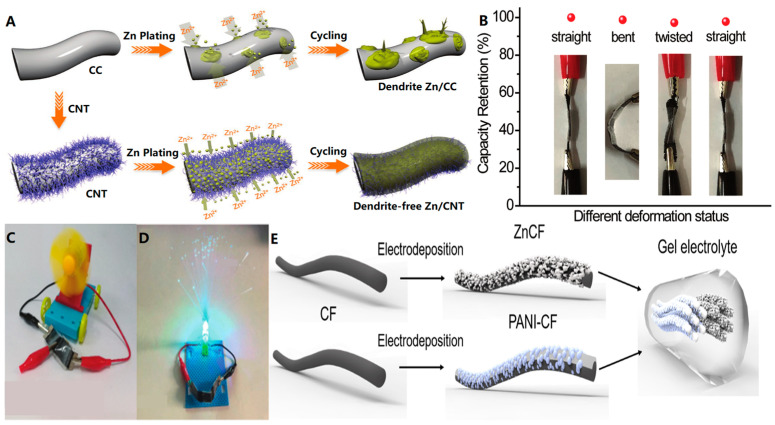
(**A**) Schematic diagram of Zn deposition on CC and CNT electrodes. (**B**) Capacity retention of quasi-solid-state Zn/MnO_2_ batteries with a Zn/CNT anode under different deformation states. (**C**,**D**) Photograph of the connected quasi-solid-state Zn/MnO_2_ battery powering the rotating fan (**C**) and LED light (**D**) [62]. Copyright 2019, John Wiley and Sons. (**E**) Schematic illustration of the fabrication of solid-state fibrous Zn/PANI batteries [63]. Copyright 2021, American Chemical Society.

In conventional zinc (Zn) batteries, the formation of Zn dendrites and “dead Zn” after multiple cycles can lead to increasing voltage hysteresis and battery failure, hindering the battery’s further application. To address this issue in Zn-ion batteries, Zeng et al. [62] utilized a combination of electrodeposition and chemical vapor deposition to fabricate a flexible three-dimensional carbon nanotube (CNT) framework as a dendrite-free solid Zn anode. The CNT framework was grown via chemical vapor deposition on flexible carbon cloth (CC), followed by Zn electrodeposition to create Zn nanosheets on the CNT surface. Compared to the original deposited Zn electrode (Figure 7A), this approach successfully eliminated the formation of Zn dendrites and other by-products. The Zn//MnO_2_ cell constructed using the Zn/CNT electrode demonstrated excellent performance (Figure 7B) and was successfully tested for powering (Figure 7C,D).

A study conducted by Cong et al. [63] investigated the inhibition of Zn dendrite growth in the design of flexible Zn cells. They utilized nanoporous Zn and polyaniline (PANI) for electrodepositing carbon nanofibers (CFs) as the anode and cathode, respectively (Figure 7E). The mechanical properties of the cells were tested, and it was observed that there was negligible capacity degradation at different bending angles (45°, 90°, 135°, and 180°), indicating the excellent flexibility of solid-state ZnCF/PANI cells. Additionally, after subjecting the device to repeated bending at 90° for 100 cycles, the capacity remained at 86.8%, demonstrating good mechanical stability. Moreover, the study successfully produced the first Zn cell using human sweat as a harmless electrolyte. They achieved this by utilizing the doping/de-doping mechanism of Cl^−^ in PANI electrodes, where a fibrous ZnCF/PANI cell was produced using artificial sweat naturally containing Cl^−^ anion as the electrolyte. This sweat electrolyte-based Zn cell shows acceptable electrochemical performance up to 145 mAh g^−1^ at 0.2 A g^−1^, opening up exciting possibilities for truly non-toxic energy storage devices. This work provides a long-life and freeze-proof fibrous battery that holds great promise for future wearable energy storage devices.

In summary, electrodeposition is a simple and practical method for preparing metal and compound layers on the surface, making it commonly used in flexible cell applications due to its ease of use and accessibility. However, one limitation of electrodeposition is the requirement for a conductive substrate, which restricts its wider application. On the other hand, chemical vapor deposition (CVD) is essential for producing highly demanding flexible substrates, as it ensures high purity and enables deposition on complex substances. However, the high equipment cost and harsh experimental environment of CVD make it unsuitable for mass production. In the future, it is anticipated that advancements in CVD technology will lead to lower equipment costs and reduced environmental requirements, allowing for its integration with methods like electrodeposition. This integration would enable wider use of deposition technology in the field of flexible manufacturing, ensuring high-performance products while maintaining cost-effectiveness.

#### 2.2.3. Printing Manufacturing

Printed manufacturing [64,65,66,67] is currently the dominant method for flexible manufacturing and is considered the most mature class of methods. It encompasses various techniques such as screen printing, gravure printing, and inkjet printing. In this process, special inks are printed on pre-prepared flexible substrates to create a flexible product. There are different types of flexible substrates available, including paper-based, PET, styrene ethylene butylene styrene (SEBS), and PDMS. Simple, flexible products, such as RF electronic tags and flexible supercapacitors, usually require a single layer of circuitry. On the other hand, complex circuits like flexible printed circuit boards (FPCBs), which are commonly used in wearable health devices, often require double-layer or even multi-layer configurations to integrate the necessary components. Print manufacturing offers several advantages, including the use of intelligent printing equipment that enables fully automated manufacturing. The accuracy of the printing process can be adjusted according to specific requirements, with market-available inkjet printers achieving nozzle accuracies of up to 20μm. Moreover, print manufacturing allows for flexibility in changing the manufacturing material of flexible devices by simply modifying the printing ink.

##### Screen Printing

**Figure 8:**
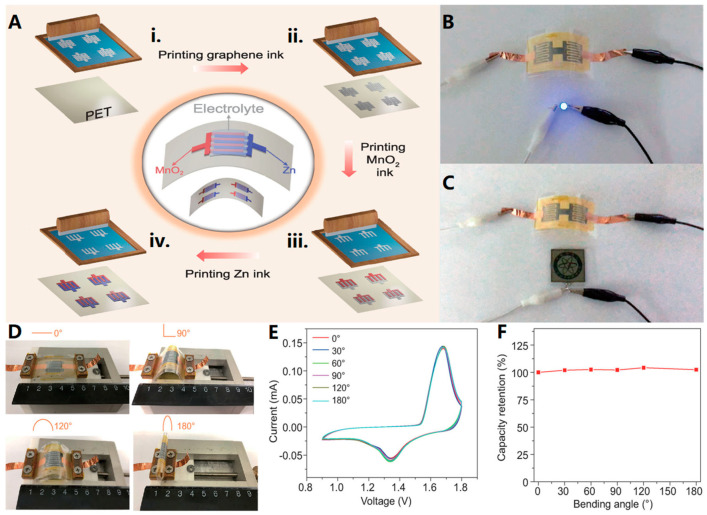
(**A**) Schematic illustration of the screen-printed fabrication of Zn//MnO_2_ planar MBs: (**i**) blank PET substrate, (**ii**) printed graphene current collector, (**iii**) printed MnO_2_ cathode, and (**iv**) printed Zn anode. (**B**) Photograph of two Zn//MnO_2_ MBs connected in series, lighting up an LED. (**C**) Powering a logo display with a flexible state. (**D**) At a scan rate of 1 mV/s, different bending angles of Zn//MnO_2_ MBs from 0° to 180° were tested. (**E**) CV curves of different bending angles. (**F**) Capacity retention at different bending angles [71]. Copyright 2019, Oxford University Press.

Screen printing [68,69,70] involves five main components: a screen printing plate, a squeegee, ink, a printing table, and a substrate. The working principle of screen printing is relatively simple: ink can pass through the mesh of the screen printing plate’s graphic section but not through the mesh of the non-graphic section. During the printing process, ink is poured into one end of the screen printing plate, and pressure is applied to the ink section using a squeegee. The squeegee moves at a uniform speed toward the other end of the plate, squeezing the ink from the graphic section onto the substrate on the opposite side, resulting in the desired printed pattern. Figure 8A provides a visual representation of this workflow. Screen printing offers several advantages, including the ability to cover a large area, produce high volume, be low-cost, have vibrant colors, and produce long-lasting prints. Nowadays, customized screen printing plates can be easily purchased at affordable prices from various online shopping platforms.

Wang et al. [71] successfully fabricated Zn//MnO_2_ planar microcells (MBs) using screen printing. Figure 8A illustrates the process of creating the Zn//MnO_2_ planar MB by printing the highly conductive graphene ink as the current collector, the Zn-based ink as the anode, and the MnO_2_-based ink as the cathode on the substrate. The MB was then packaged. This method yielded a Zn//MnO_2_ planar MB with suitable ink rheological properties, high conductivity of the microelectrodes, and impressive performance characteristics. The MB exhibited a high bulk energy density, excellent safety, long-term cycling stability (83.9% capacity retention after 1300 cycles at a high rate of 5C), and superior flexibility. In addition, the functionality of the MBs was demonstrated in Figure 8B,C, where the MBs successfully powered a light-emitting diode (LED) for an extended period even when bent. Furthermore, the MBs were able to illuminate a display screen. Figure 8D–F shows that all cyclic voltammetry (CV) curves of MBs overlapped under different bending angles, with almost 100% capacity retention even at a bending angle of 180°.

**Figure 9:**
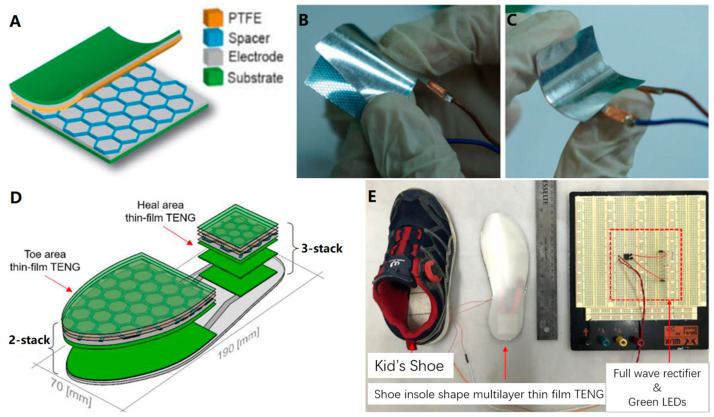
(**A**) 3D structure diagram of thin-film TENG. (**B**,**C**) Photograph of a single thin-film TENG. (**D**,**E**) Insole MT-TENG: (**D**) was a schematic diagram of the structure of the insole generator using MT-TENG; (**E**) was the setting of the insole generator [72]. Copyright 2018, John Wiley and Sons.

Screen printing technology, although simple to operate, has extensive applications beyond just preparing flexible electronic circuits and flexible energy storage devices. It can be utilized for a wide range of diverse designs. For instance, Hong et al. [72] employed this technique to design and fabricate a multilayer thin-film turboelectric nano-generator (MT-TENG) that possesses self-generating capabilities and can be integrated with microelectronic systems. When combined with flexible wearable devices, it can cater to specific application requirements and provide a stable long-term power supply. This device exhibits exceptional electrical performance and mechanical stability (Figure 9A–C). To illustrate its effectiveness, the device was incorporated into the insole of an athletic shoe. When an 8-year-old boy wore the shoe and walked, the open-circuit voltage reached 20 V, effectively illuminating nine light-emitting diodes connected in series (Figure 9D,E).

Screen printing has gained widespread use in industrial production and scientific research due to its low cost and simple process. However, these advantages also limit its printing accuracy, making it suitable only for designs that require low printing accuracy, simplicity, and low complexity, such as simple circuits and supercapacitor preparation. While simple circuits offer flexibility and stretching capabilities, they are limited in their ability to integrate complex electronic components, thus limiting their functionality. Flexible energy storage devices prepared through screen printing have achieved flexibility in bending but still lag behind traditional rigid batteries in terms of overall stability and performance. This is a common challenge for all flexible energy storage devices. At present, screen printing has found sufficient applications, but improved accuracy while maintaining low costs and easy operation would undoubtedly open up new opportunities for its development.

##### Gravure Printing

**Figure 10:**
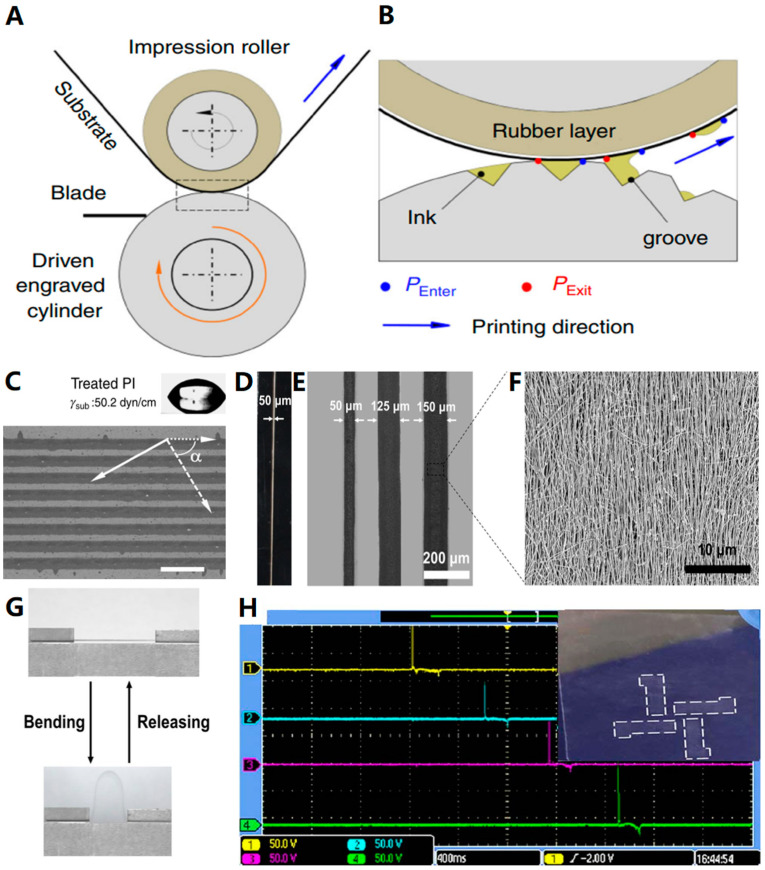
(**A**,**B**) Schematic of roll-to-roll gravure printing employed by Shin et al.: (**A**) general configuration and (**B**) detailed view of contact points. (**C**) An optical image of a line pattern printed with rotogravure at a printing angle of 60° and a printing speed of 10.5 m/min. Dotted lines and continuous lines represent directions perpendicular to and consistent with the printing direction, respectively; the surface energy of the treated PI substrate was 50.2 dyn/cm; the scale bar represented 500 μm [78]. Copyright 2016, Springer Nature. (**D**) Photograph of printed AgNW with a length of 3 cm and a width of 50 μm. (**E**) Optical images of three AgNWs with different line widths (50, 125, and 150 μm) and the same spacing of 100 μm. (**F**) Morphology of printed AgNW. (**G**) Photographs of the bending test process [79]. Copyright 2018, Springer Nature. (**H**) Screenshot of a four-channel Tektronix MSO2024 oscilloscope with a signal of about 80 V. The inset is a picture of the printed AgNW array (mainly 4 mm*12 mm, about 2.3 kΩ/sq sheet resistance, and 95.6% transparency) [80]. Copyright 2008, IOP Publishing, on behalf of the Japan Society of Applied Physics (JSAP).

Gravure printing [73,74,75,76,77] is one of the four major printing methods, alongside screen printing, letterpress, and lithography. The principle of gravure printing involves coating the entire surface of the printing plate with ink and then using a special scraping mechanism to remove the ink from the blank part. This leaves the ink only in the screen cavity of the graphic part; with greater pressure, the ink is transferred to the surface of the substrate, resulting in a printed product (Figure 10A,B [78]).

Gravure printing is a direct printing method where the graphic part of the printing plate is concave, with varying depths corresponding to the image levels. The blank part of the plate is raised and on the same plane. Due to the recessed graphic portion, the ink in the finished print appears raised, making this method suitable for printing securities or currency. The advantages of gravure printing include its wide range of paper applications, strong color expression, and resistance to wear and tear. However, it has some drawbacks, such as expensive plate-making and printing costs, and it is not suitable for small print runs. While gravure printing may not become the mainstream method for flexible electronic preparation, it still plays a crucial role in certain specialized processes. As one of the four major printing methods, gravure printing holds a pivotal position in flexible manufacturing.

Gravure printing stands out as an optimal choice for preparing resistant and thicker patterns in specialized applications, even though other flexible electronic production processes can generally fulfill flexibility requirements. Shin et al. [78] successfully prepared thick-film silver electrodes via the roll-to-roll gravure printing technique. Figure 10C illustrates the obtained patterned silver electrodes, which featured a length of 1 m, a line width of 121+/−2 μm, an average thickness of 6.5 ± 2.2 μm, and a resistivity of 9 μΩ·cm. This demonstration highlights the potential of gravure printing technology in manufacturing large-area, high-fidelity thick-film flexible printed circuit boards at a high production scale.

In a separate study, Huang et al. [79] explored the utilization of gravure printing for the fabrication of flexible electronic devices employing aqueous silver nanowires (AgNWs) ink. As shown in Figure 10D–F, the AgNWs have high resolution and high conductivity, with a resolution as fine as 50 μm and conductivity as high as 5.34 × 10^4^ S cm^−1^ after completion of gravure printing on a flexible PET substrate. Additionally, the pattern exhibited exceptional flexibility even after undergoing repeated bending, as illustrated in Figure 10G. Notably, the resistance of the pattern did not experience an increase even after undergoing hundreds of bending cycles. This experimental investigation unequivocally proves that the integration of AgNWs with gravure printing holds substantial promise for commercial applications within the field of printed and flexible electronics.

We previously discussed the use of screen printing for the fabrication of triboelectric nanogenerators (TENGs), and Peng et al. [80] have also designed TENGs using a gravure printing method. In their study, they employed fluorinated ethylene propylene (FEP) films with intaglio-printed AgNW patterns as transparent electrodes for three-phase electrical sensors. Figure 10H demonstrates the continuous tapping of the back side of the sensor with a gloved finger while simultaneously measuring the output voltage using a four-channel oscilloscope. The results confirmed that the FEP with AgNW electrodes generated an output voltage ranging from 70 to 80 V. This research introduces a novel and efficient approach for the fabrication of transparent triboelectric sensors.

Gravure printing is capable of fulfilling process requirements and can, to some extent, replace other printing methods. Although it has notable drawbacks, such as high plate-making costs and unsuitability for small batch production, its simplicity, vibrant color expression, and durability make it advantageous. Additionally, it can be utilized for special products like securities or currency, allowing it to maintain a presence in the flexible printed electronics market. To further expand its potential in scientific experiments, commercial production, and everyday design, reducing the plate-making costs associated with gravure printing would enable the production of a wider range of flexible products.

##### Inkjet Printing

Inkjet printing technology [81,82], also known as direct deposition manufacturing technology, involves the ejection of small droplets of ink onto a substrate through a nozzle. This method offers the advantages of high automation and exceptional printing accuracy, enabling the achievement of micron-level printing and manufacturing. In comparison to screen printing and gravure printing, inkjet printing surpasses them in precision. However, the process requires meticulous attention to ink parameters and properties, as any discrepancies can lead to clogging issues, thereby complicating equipment maintenance and subsequent experiments.

**Figure 11:**
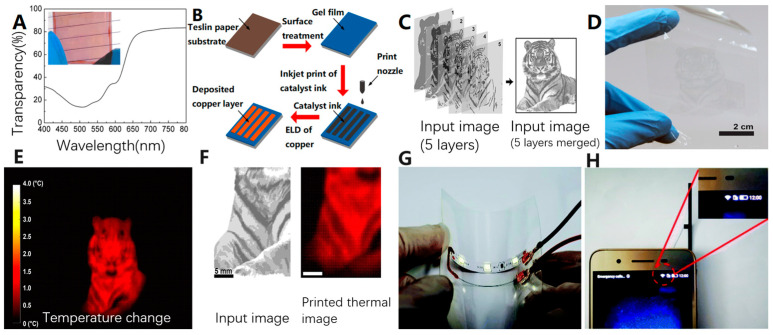
(**A**) Transparency of the electrode printed seven times with the picture in the inset [83]. Copyright 2015, AIP Publishing. (**B**) A general scheme for the fabrication of flexible electronic circuits on paper substrates by inkjet printing combined with ELD [91]. Copyright 2016, American Chemical Society. (**C**) Raw input image file for GNR images. (**D**) Thermal plasma GNR image inkjet printed on flexible FEP film. (**E**) Thermal image (temperature change extracted from baseline) recorded by an infrared camera when illuminated with NIR light on a printed thermal plasma image. (**F**) Enlarged thermal image in (**E**) and corresponding input image in (**C**). Dark spots in the input image appeared brighter in the thermal image because higher GNR densities led to larger temperature changes [92]. Copyright 2018, American Chemical Society. (**G**) The printed wire successfully illuminated the LED lamp, and at the same time, the whole circuit was subjected to bending. (**H**) The WiFi signal was restored after the smartphone was connected to the rGO antenna [94]. Copyright 2021, Royal Society of Chemistry.

Lu et al. [83] described a method for inkjet printing transparent top electrodes in their paper. They utilized an inkjet printer to print conductive silver paste on a glass substrate that had been spin-coated with a transparent conductive network [84]. This conductive network served as the top electrode for an inverted translucent organic photovoltaic (OPV) device [85] (Figure 11A). To improve the film quality, the glass substrate was spin-coated with PEDOT: PSS: MoO_3_, which acted as an anode buffer layer. Moreover, they addressed the issue of nozzle clogging during inkjet printing by incorporating high-boiling-point glycol into the AgNW ink, reducing ink evaporation. Notably, as the number of prints increased, the sheet resistance of the printed silver nanowires decreased significantly, leading to excellent electrical properties.

However, inkjet printing of silver nanoink also presents a common and critical issue. To prevent nozzle clogging and improve ink performance, various reagents, such as wetting agents, defoamers, and dispersants, are often added to the printing ink. Unfortunately, these additional reagents can negatively impact the conductivity of the printing wires. Furthermore, the silver element in the ink exists in the form of nanoscale particles or rods, resulting in a lack of connectivity in the initial printing pattern on the substrate. Consequently, to enhance the electrical conductivity of the silver ink circuit pattern, a subsequent heating and drying process is necessary. This process evaporates the reagent components that affect conductivity and melts the nanoscale silver particles or wires, allowing them to reconnect and polymerize, thereby improving overall electrical conductivity. However, the preparation process increases the complexity and time required for inkjet printing, and the heating and drying can cause irreversible damage to the flexible substrate. For instance, the sintering temperature for typical silver nanoparticles is around 150 °C (the melting point of silver is 961.8 °C). To obtain better sintering effects, higher temperatures (200 °C and above) are necessary [86,87,88,89,90]. Unfortunately, many flexible substrates cannot withstand prolonged exposure to these high temperatures. Commercial PET films can tolerate temperatures of up to 150 °C for short periods but exhibit noticeable discoloration and deformation when exposed to temperatures of 150 °C or higher for several hours. Consequently, these challenges must be addressed when considering the subsequent printing of silver ink patterns.

Wang et al. [91] developed a simpler and more cost-effective method for inkjet print manufacturing. This method utilizes a silver-based catalyst ink and a flexible paper-based substrate. The process involves surface-treating the paper substrate to create a gel film that prevents ink penetration. Next, a specific pattern is inkjet printed on the substrate using the catalyst ink. Copper elements are then deposited onto the printed pattern through metal electroless deposition (ELD) (Figure 11B). This approach offers several advantages over conventional silver solution printing patterns. The deposited copper elements ensure a conductivity of up to 70% of bulk copper, eliminating the need for sintering and heat treatment of the silver solution circuit in the conventional process.

Kang et al. [92] introduced a novel approach for creating thermal plasmonic patterns using inkjet-printed gold nanorod (GNR) inks. This method enables selective modification of neuronal network activity and allows for the development of high-quality, biocompatible thermal plasmatic interfaces on various types of substrates (rigid/flexible, hydrophobic/hydrophilic). The inkjet-printed GNR images on the flexible fluorinated ethylene-propylene substrate (FEP film) exhibited exceptional fidelity, as shown in Figure 11C–F. By utilizing near-infrared (NIR) light irradiation, the printed thermal plasma patterns were able to selectively heat and modulate neuronal activity. These products can be easily integrated with transparent and implantable neural interface devices, offering the ability to record neural signals or modulate neural activity. This experiment not only provides new methods for research in the field of neural engineering but also opens up possibilities for flexible product applications and biointegration [93].

Of course, in addition to using expensive gold and silver solutions to print flexible electronic patterns, researchers are actively exploring alternative methods to reduce costs. Lv et al. [94] utilized graphene printing [56,95,96,97,98], a more affordable option, to fabricate flexible circuits and radio frequency (RF) antennas. Unlike gold and silver solutions, graphene printing material does not require the addition of stabilizers to disperse them, thereby avoiding any adverse effects on circuit conductivity. Furthermore, the heating treatment temperature after printing is limited to approximately 70 °C, preventing any damage to the flexible substrate from high-temperature exposure. Initially, graphene oxide (GO) films derived from GO suspensions are insulating, but they can be reduced to dense graphene films, exhibiting excellent electrical conductivity. Building upon this, Lv et al. employed a piezoelectric on-demand inkjet printer for reactive inkjet printing, where two reactive components are printed on top of each other to trigger a reaction and form a product. Figure 11G shows the conductivity and mechanical durability tests of the printed reduced graphene oxide (rGO) circuit. It can be seen that the rGO circuit prepared by reactive inkjet printing can successfully light up the LED light bar and does not affect its performance even under bending. Figure 11H shows the graphene RF antenna prepared by the same method, and the cell phone WIFI signal is restored after connecting the smartphone WIFI contact point.

The use of inkjet printers is widely prevalent in our daily lives, especially for printing and copying paper materials. However, as mentioned earlier, inkjet printing devices have extremely high requirements for printing inks, including viscosity, pH, and surface tension, to prevent damage to the pipeline and print head. After printing, the equipment must also be promptly and repeatedly cleaned to prevent nozzle clogging. Moreover, conditioning reagents added to the ink necessitate additional operations such as cleaning and heat treatment to achieve properties in the printed products. These tasks not only consume significant energy and time for researchers but also pose challenges for practitioners in the field of inkjet printing. While improvements in inkjet printing inks can partially mitigate these issues and be integrated with other processes, they also complicate the manufacturing process and increase preparation time. Therefore, it is crucial to explore further improvements in the inkjet printing process beyond ink formulation enhancements.

Inkjet printing offers numerous advantages, including fast deposition, high resolution, simplified procedures, and non-contact direct printing. To address existing challenges, several aspects can be considered. First, improving ink performance and streamlining its integration with other production processes can be explored. Additionally, optimizing key components of high-precision inkjet printing equipment, such as pipes and nozzles, reduces the requirements for printing ink parameters and equipment maintenance, thereby enhancing equipment durability and adaptability. This would enable high-precision inkjet printing devices to operate with greater independence from ink quality, similar to office printers. Consequently, these devices would find wider development opportunities not only in flexible electronic printing and manufacturing but also in other fields, such as personalized, customized textile printing, office printing, advertising imaging, airbrushing, packaging, label printing, and architectural design. In industrial digital printing, high-precision inkjet printing equipment can facilitate higher-quality design and production. It is particularly useful during the flexible transformation of traditional rigid PCBs, as it can easily resolve welding issues caused by the high integration of circuit modules and high-precision chip pins.

#### 2.2.4. Etching

The etching process is a widely used material treatment method in scientific research and commercial production [36,37,57,99,100,101]. One particular technique that has garnered significant attention in recent years is photolithography. This method involves coating the substrate with a photoresist and exposing it to ultraviolet (UV) light through a photomask. The irradiated area is then dissolved away by a developer, allowing for selective etching or ion implantation of the substrate. Once the process is complete, the photoresist is removed, leaving behind a specific product that can be used as a basis for subsequent product design. This substrate etching method, in conjunction with photomasks, is mostly used in the preparation of integrated circuits, ranging from traditional silicon-based circuit boards to miniaturized nanoscale chips.

The described preparation procedure is highly adaptable for processing flexible substrates, allowing for a range of simple or complex processes depending on the desired accuracy of the final product. To illustrate, when preparing basic wires or patterns on a flexible substrate, a printing process can be used to apply etch-resistant ink to the substrate surface, forming a pattern mask. Subsequently, a simple etching solution is employed to etch the unmasked area, followed by the removal of the etch-resist ink to obtain the desired flexible pattern. This experimental substitution of photoresist with etch-resist ink eliminates the need for a developer soak. Various flexible substrates can be chosen, such as copper-clad PET film or copper-clad PI film, for the preparation of simple flexible integrated circuits. Using this method, commercially available flexible copper circuit patterns can be easily fabricated. Etching technology finds extensive applications in the fabrication of flexible devices. For example, Marques et al. [102] utilized inkjet printing and etching to design multilayer flexible devices; Wang et al. [51] designed stretchable transistor arrays for skin electronics based on etching processes and copper mask protection strategies to effectively pattern stretchable semiconductors without compromising their electrical properties.

**Figure 12:**
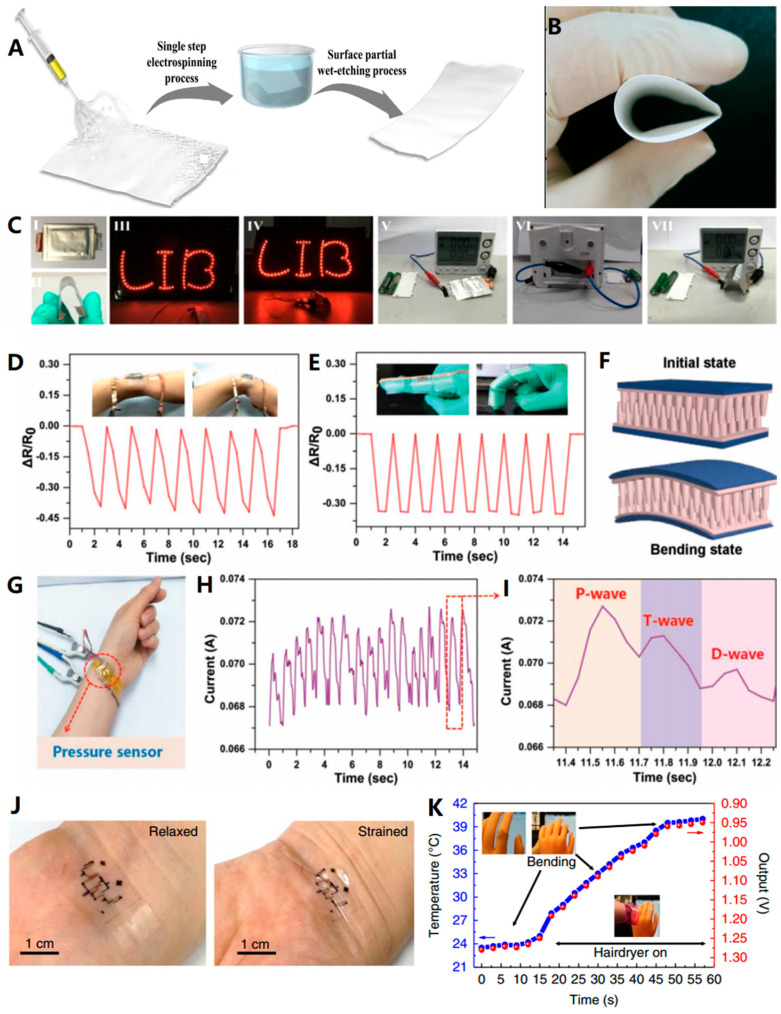
(**A**) Schematic diagram of the fabrication process of flexible zeolite fibers with high surface area based on core-shell structure. (**B**) Optical image of a zeolite fiber filter with a diameter of 7 cm [37]. Copyright 2018, Elsevier. (**C**) Optical images of prepared all-flexible LIB (**I** and **II**) lit up “LIB plate” (**III**,**IV**) and “stopwatch” (**V**–**VII**) in the flat and bent positions, respectively [99]. Copyright 2016, Elsevier. (**D**,**E**) Response curves of the pressure sensor during wrist bending/releasing and finger bending/releasing, respectively. (**F**) Schematic illustration of micropillar deformation during sensor bending. (**G**) A digital photo of the sensor attached to the wrist. (**H**,**I**) The human body’s electrocardiogram is detected by the pressure sensor fixed to the wrist. (**I**) showed one of the enlarged waveforms, which could accurately detect and clearly distinguish three typical peaks, namely, percussion wave (P waves), tidal wave (T waves), valley, and diastolic wave (D waves). P and T waves were related to blood pressure, while D waves represented heart rate [101]. Copyright 2019, John Wiley and Sons. (**J**) An optical photograph showing the consistency of the temperature sensor. When the wrist is bent, the sensor is laminated to the skin. (**K**) Demonstration of a stretchable temperature sensor. The illustration shows a sensor attached to the knuckle of a flexible rubber prosthesis, showing stable functionality during repeated finger bending [103]. Copyright 2018, Springer Nature.

Figure 12 illustrates various etching methods that can be effectively used in the production of flexible materials. For instance, Kim et al. [37] used the electrospinning method to prepare flexible zeolite fibers with a core-shell fiber structure. Subsequently, a wet etching process was used to partially etch the fiber surface, resulting in enhanced strength and surface area (Figure 12A). Balogun et al. [99] thermally etched commercial carbon cloth (CC) to significantly improve the lithium storage properties, enabling its direct use as electrode material for both half-cells and all-flexible full lithium-ion batteries (LIBs). The excellent performance of LIB prepared via this method is demonstrated in Figure 12D. In addition, Zhang et al. [101] fabricated highly sensitive, cost-effective flexible pressure sensors with micropillar arrays for wearable electronics via a novel metal-assisted chemical etching process. The tests shown in Figure 12E–J further validate the immense potential of this product in applications such as wearable electronic skin, soft robotics, and healthcare monitoring devices. Zhu et al. [103] combined photolithography with other processes like spin coating and screen printing to prepare a temperature sensor with a strain suppression function (Figure 12J,K). This sensor, based on carbon nanotube transistors, exhibits stretchability and stable electrical output, with a measurement error of only +/−1 °C. As shown in Figure 12K, the sensor is attached to the knuckle of the flexible rubber prosthesis, and the sensor shows stable functionality during repeated finger bending.

However, the preparation of fine patterns, especially the use of photolithography to produce nanoscale chips, poses significant challenges in terms of process accuracy and equipment costs. Only a few manufacturers worldwide have mastered this advanced technology, highlighting its technical difficulty. Photolithography is known as the most precise technique for deposition and is often regarded as the pinnacle of the modern optical industry. Therefore, owing to its technical difficulty and production cost, the application and widespread adoption of photolithography will be restricted. This limitation is particularly evident in the field of flexible manufacturing, where even simple etching methods are more commonly employed. Therefore, there is still a long way to go in the development of etching technology, particularly in overcoming the challenges with photolithography. The crucial focus for future progress lies in effectively balancing etching accuracy and cost.

#### 2.2.5. Laser

Laser etching is a well-established technique that uses a high-energy pulsed laser beam to create precise patterns on material surfaces through the etching of small, narrow grooves. This process is widely utilized in electronics manufacturing, particularly for circuit pattern etching on substrates, due to its numerous advantages, such as high precision, automated manufacturing, and superior etching results. However, the application of laser technology in flexible manufacturing extends beyond this principle. Depending on the application and development of the laser, there are already a variety of flexible product creation solutions based on laser technology [61,95,96,97,104,105,106,107,108,109,110].

**Figure 13:**
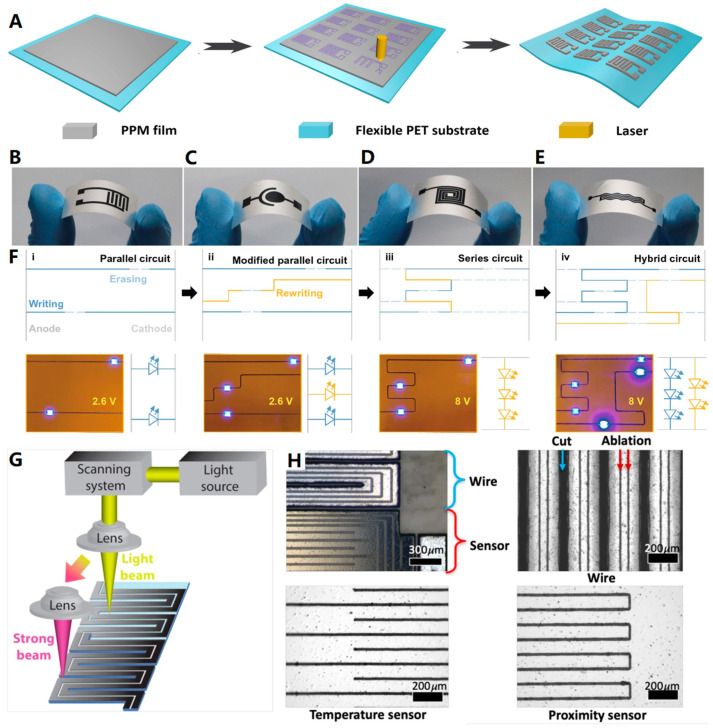
(**A**–**E**). SSCs with high electrochemical performance from PPM: (**A**) Schematic diagram of the fabrication process of coplanar SSC electrode patterns; SSC electrode patterns with (**B**) interdigitated, (**C**) concentric circular, (**D**) concentric rectangular, and (**E**) wave-shaped configurations [111]. Copyright 2020, Elsevier. (**F**) Schematic illustration of the pattern changes of the reprogrammed circuit and its corresponding actual digital image. Subsequent circuits were gradually modified from the first case, but on different PI substrates. The LEDs were introduced after completing these circuits to confirm that they were conducting [112]. Copyright 2021, Springer Nature. (**G**) Illustration of the laser ablation strategy, using low power to trace metal traces and high power to cut PET films. (**H**) Microscopic image of the sensor and wires, showing the ablated aluminum sensor (red) and cut interconnect wires (cyan) [113]. Copyright 2022, Springer Nature.

In 2020, Chu et al. [111] prepared an ultra-thin, robust polymer film for wearable solid-state electrochemical energy storage. They employed a simple blade coating to process a hybrid solution onto a glass substrate, resulting in the production of a self-contained polyaniline/polyvinyl alcohol membrane (PPM) with a refined cross-linked structure. This PPM was tightly attached to a flexible PET film as substrate and further cut into pre-designed patterns using a UV laser beam to fabricate solid-state supercapacitors (SSCs) (Figure 13A). Figure 13B–E shows individual SSCs with various structures, indicating that PPM has good processability in laser fabrication. Moreover, these SSCs based on PPM exhibited outstanding electrochemical properties.

Zhou et al. [112] conducted research that expanded the application of laser technology in flexible manufacturing. They demonstrated the ability to write, erase, and rewrite highly conductive copper patterns on flexible PI substrates using a low-cost liquid precursor and a laser. By scanning a focused laser over a specific area, a copper pattern can be created, and the pattern can be selectively erased by irradiating the out-of-focus laser. The durability of the pattern was tested through 3000 bending cycles, and the relative resistance change was found to be approximately eight times greater than its initial one. Figure 13F shows a lit LED test of the prepared pattern. The circuit design and changes in the test are all using the laser write, erase, and rewrite technique, illustrating the great potential of the pattern prepared based on this technique for functional design applications in electronics.

Additionally, laser technology can be easily applied to flexible metalized films that are readily available on the market. Ham et al. [113] utilized UV laser ablation and cutting to pattern temperature sensors and proximity sensors on aluminized PET films. By controlling parameters such as laser power, they were able to ablate the metal without damaging the underlying PET material. Furthermore, by cutting the film in a serpentine Kirigami structure [114], highly stretchable interconnects were created (Figure 13G,H). This approach enables the development of stretchable multi-modal sensor networks for soft-robot interaction, opening up possibilities for applications such as food handling and human-soft-robot interaction.

Despite being used in various fields since their introduction in the last century, lasers still face limitations in flexible manufacturing. One main reason is the high cost of laser equipment, which makes it less cost-effective. Moreover, laser technology lacks versatility in manufacturing flexible products, which often require pattern ablation or inducing chemical reactions in solutions on flexible substrates. These limitations not only hinder potential applications of lasers but also restrict their role to assist rather than dominate the flexible manufacturing process. However, it is important to note that laser technology possesses mature characteristics such as high precision and automation. As research progresses, we can anticipate further innovations and designs in the field of flexible manufacturing that will enhance the capabilities of laser technology.

### 2.3. Preparation of Flexible Energy Storage Devices Based on Flexible Electrolytes

**Figure 14:**
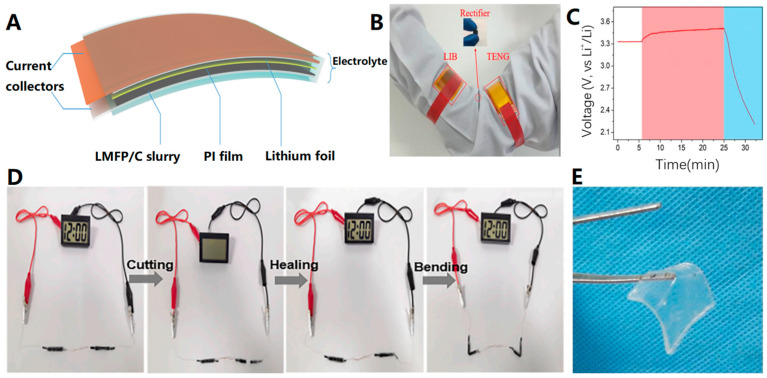
(**A**) Schematic illustration of the flexible LIB battery. (**B**) A photo of the wearable self-charging power pack composed of a flexible battery and flexible TENG. (**C**) Charge and discharge curves of the flexible LIB. The operator rested in the white area. As the operator’s arm moved, the battery charged in the pink area. The battery was discharged in the blue area [125]. Copyright 2018, Elsevier. (**D**) Digital photographs of two batteries connected in series being cut/healed and bent by self-healing treatment to power a displayer [123]. Copyright 2023, Elsevier. (**E**) Optical image of flexible water-deactivated polyelectrolyte hydrogel electrolyte [124]. Copyright 2018, John Wiley and Sons.

As a crucial aspect of flexible electronics, the importance of flexible energy storage [115,116,117,118,119,120,121] is undeniable. However, research on flexible batteries still predominantly relies on traditional battery design. Merely enhancing the flexibility of components such as electrical-grade materials and fluid collectors is insufficient in the design process. To achieve greater flexibility, it is imperative to utilize flexible electrolyte materials [122,123,124] in constructing energy storage devices. This approach enables the construction of batteries with enhanced flexibility. For instance, Li et al. [125] investigated an MMA/PI-based quasi-solid electrolyte to prepare flexible LIBs by completely polymerizing methyl methacrylate MMA on a PI substrate (Figure 14A). This electrolyte not only offers a wide electrochemical window but also demonstrates superior performance compared to conventional liquid electrolyte button cells. Additionally, it was also integrated with a flexible triboelectric nanogenerator (TENG) to realize a self-powered battery pack for wearable energy supply (Figure 14B,C).

The impact of flexible electrolytes on energy storage goes beyond their initial applications. For instance, Yang et al. [123] prepared a double-cross-linked polyampholyte hydrogel electrolyte with high mechanical strength and excellent ionic conductivity. This electrolyte demonstrated multiple repeated healing abilities under various conditions. Zn-ion batteries (ZIBs) assembled with this electrolyte exhibited excellent electrochemical performance and multifunctional healing properties. Moreover, the fractured/healed ZIBs had superior flexibility and stable fracture/healing cycles over six cycles. Figure 14D also illustrates that this hydrogel electrolyte could extend the application of flexible energy storage devices to other fields. In addition, Wei et al. [124] utilized water-deactivated polyelectrolyte hydrogel electrolytes (Figure 14E) in flexible high-voltage supercapacitor applications. The resulting supercapacitors demonstrated both flexibility and customizability, providing a new avenue for designing high-energy-density energy storage devices for flexible wearable electronics.

For the flexible design of electrolytes, the primary focus is flexible energy storage devices, as traditional energy storage devices cannot meet the needs of flexible electronic devices. The recent trend of using liquid electrolytes in flexible battery designs poses issues such as instability and leakage. In addition, other forms of flexible energy storage devices, like forked finger electrodes and supercapacitors, can only supply energy to low-power devices such as small LED lights. Therefore, it is crucial to study the design of flexible electrolytes. The flexible treatment of solid electrolytes not only meets the flexibility requirements of energy storage devices but also offers improved stability compared to liquid electrolytes. By optimizing the design of flexible electrolytes, the overall performance of energy storage devices can be further enhanced. Thus, future research should focus on investigating more materials for flexible electrolytes and improving their performance to develop various types of flexible batteries. This will enable the realization of complete and true flexibility in energy storage devices and better serve the energy supply needs of flexible electronic devices.

## 3. Stacking Process: Complex Multilayer Flexible Device Preparation

The flexibility of electronic devices is a crucial aspect of the advancement of technology. However, current research in flexible electronics primarily focuses on single-layer circuits, which hinders the development of small-area devices with special requirements. This limitation prevents the integration of multiple functionalities on the device, greatly restricting its practicality. Therefore, it is essential to prioritize the research and design of multilayer flexible electronic devices [126,127,128,129,130] to overcome these limitations and enhance their functionality.

**Figure 15:**
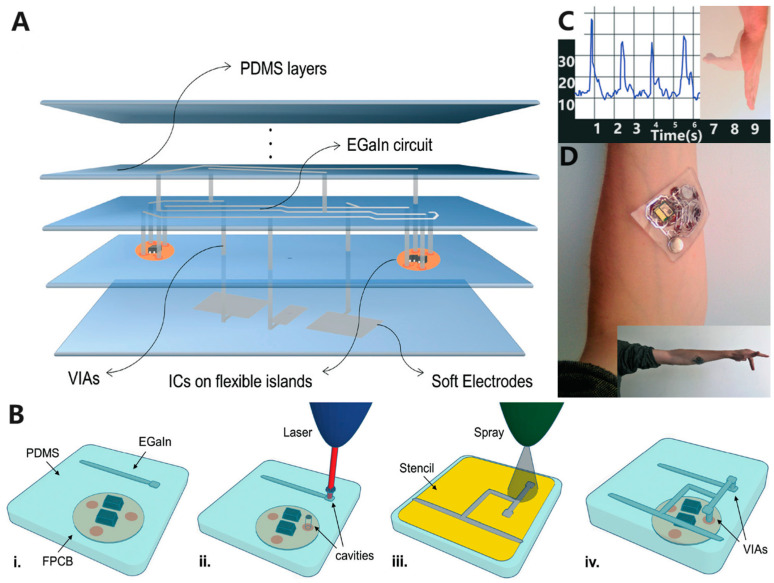
(**A**) A disassembled view of a multilayer stretchable circuit with interconnected microelectronics and flexible islands with soft electrodes on the bottom layer. Additional layers and VIAs can be fabricated. (**B**) Production of VIAs: (**i**) The flexible printed circuit board and GaIn interconnects were encapsulated in a stretchable elastomer; (**ii**) The selected traces and pads were accessed by vertical laser ablation; (**iii**) When spray coating on a patterned stencil, the cavities were filled with liquid metal; (**iv**) The patterned stencil was removed before encapsulation of VIAs and new circuit layers. (**C**) EMG signals from repeated hand gestures were transmitted to a computer application. (**D**) Fully integrated EMG patch on the forearm (inset: adhesion of the EMG patch to the skin during hand gestures) [102]. Copyright 2019, Royal Society of Chemistry.

The preparation of multilayer devices typically involves multiple complex processes, with a crucial focus on achieving an effective and reliable electrical connection between the layers. Marques et al. [102] successfully prepared multilayer stretchable hybrid circuits using a combination of inkjet printing, metal etching, laser ablation, coating, liquid metal (LM)-filled vertical interconnect accesses (VIAs), and spray plating [38,110,131,132]. Figure 15A illustrates the principal disassembly diagram of their approach. In the experiments, circuit patterns were formed on the copper surface by inkjet printing. The printed patterns were then subjected to an acidic solution to etch the exposed copper, resulting in the formation of a flexible printed circuit board (FPCB). The FPCB was used to carry solder chips and was placed between two layers of PDMS.

The liquid metal used in this study was eutectic gallium-indium (EGaIn), which is a widely used material. Multilayer flexible circuits and VIAs were prepared by spraying EGaIn and depositing them with patterned stencil masks. Laser ablation was employed to create cavities in the closed PDMS, which were filled with EGaIn to form VIAs. These VIAs allowed for the connection of two or even multiple layers of flexible circuits (Figure 15B). The copper pads of the FPCB were used to bridge the connection between the VIAs and the underlying flexible circuit. The circuit prepared using this method exhibited excellent flexibility, withstanding over 80% strain. Additionally, there were no significant changes in the electrical properties of the interface or leakage of liquid metal even after 1000 loading cycles. The researchers also developed various applications based on this technology, including a stretchable touchpad, a pressure detection film, and a fully integrated multilayer electromyography (EMG) circuit patch (Figure 15C,D). The EMG circuit patch, measuring 47 × 57 × 2.7 mm, was capable of measuring muscle activity and detecting human gestures using soft electrodes in contact with skin. Overall, this experimental method effectively combines multiple processes and provides a feasible approach for preparing multilayer flexible electronic devices. It also expands the design possibilities for EGaIn materials.

**Figure 16:**
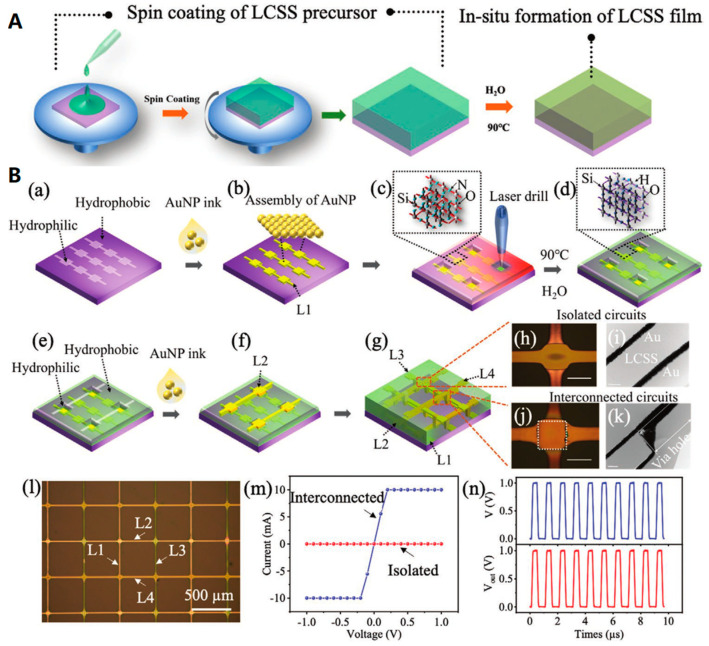
(**A**) Schematic illustration of LCSS films spin-coated at low temperatures under humid conditions. (**B**) Three-dimensional integration of multilayer AuNP conductive circuits. (**a**) LCSS surface preprinted with hydrophobic and hydrophilic regions by VUV exposure; (**b**) First layer (L1) electronic circuit by printing conductive AuNP ink; (**c**) Creating holes in the soft PHPS precursor film by laser drilling; (**d**) Formation of solid LCSS films by cross-linking of precursor PHPS at 90 °C; (**e**) Hydrophilicization of the LCSS surface by VUV exposure for printing the second layer (L2); (**f**) L2 was printed with AuNP ink, and L1 and L2 were connected to each other via holes; (**g**) Integration of four-layer AuNP circuits; (**h**–**k**) Optical micrographs (**h**,**j**, scale bar: 50 μm) and cross-sectional TEM images (**i**,**k**, scale bar: 200 nm) of isolated and interconnected circuits; The dashed box marked in (**j**) represented the implanted AuNP connection in the laser-drilled via hole, with a size of 50 × 50 µm2; (**l**) Optical diagram of the four-layer AuNP circuit, where L3 and L4 denote the third and fourth layer AuNP circuits; (**m**) I–V measurements of interconnected (dashed blue lines) and isolated (dashed red lines) circuits with applied voltages from −1 to 1 V and compliance current set to 10 mA; (**n**) Transient response of the input voltage (blue trace) to the output voltage (red trace) in an interconnect circuit with the applied voltages of 1 V within 1 µs [133]. Copyright 2021, John Wiley and Sons.

Sun et al. [133] explored a layer-by-layer printing strategy for high-performance flexible electronics. They utilized low-temperature catalyzed solution-processed SiO_2_ (LCSS) to fabricate multilayer electronic devices, including three-dimensional conductive circuits and thin-film transistors (TFTs). The SiO_2_ dielectric precursor used was spin-coated perhydropolysilazane (PHPS). By catalyzing the transformation of PHPS at low temperatures, highly transparent, bendable SiO_2_ LCSS films were obtained. To achieve selective deposition, the researchers employed vacuum ultraviolet (VUV) treatment and gold nanoparticles (AuNPs) ink. The VUV irradiation induces different wettability on the LCSS surface, allowing for the selective deposition of AuNP ink and the formation of circuit patterns. Circuit substrates and the septa between the multilayers are LCSS films prepared by a layer-by-layer printing strategy. Laser drilling was used to create holes between the layers, and the same AuNP ink was injected for electrical connection. This process resulted in the creation of a multilayer, three-dimensional conductive circuit through layer-by-layer printing and laser drilling (Figure 16A,B). The prepared circuit showed a resolution of up to 10 μm with good shape, high fidelity, and sharp edges. This fully printed technology enables low-cost, large-scale, and environmentally friendly manufacturing of high-performance TFTs compared to conventional methods. The combination of low-temperature catalytic processing and full printing opens up new possibilities for the future fabrication of flexible electronic devices, particularly stacked multilayer circuits.

**Figure 17:**
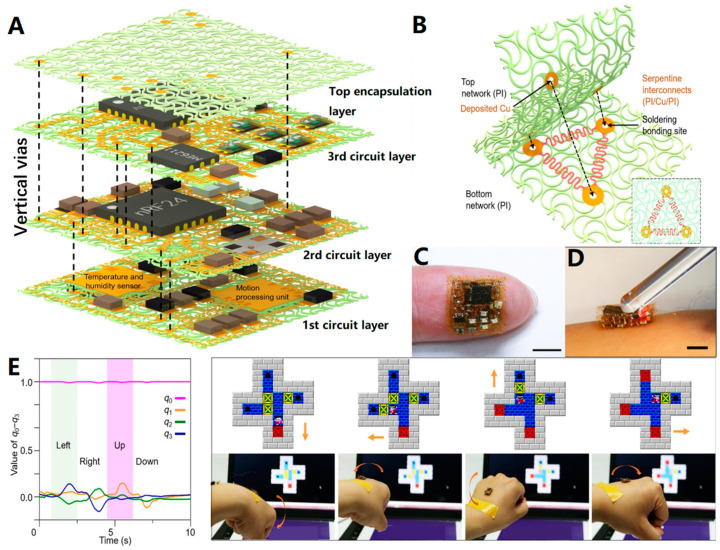
Design and fabrication of densely packed, stretchable electronic systems based on SMNMs. (**A**) Disassembly diagram of the device system. The system contained 42 integrated circuits with lateral dimensions of ~11.2 mm × 10.1 mm and area coverage of ~110%. Sub-circuits were arranged and electrically connected through vertical vias, highlighted with dashed lines. (**B**) Process of integrating and encapsulating serpentine interconnects with bilayer network materials. (**C**) Photographs of the SMNM-based stretchable electronic system mounted on the index finger. (**D**) Deformation of the device system when pressed (~15%). (**E**) Demonstration of the functional characteristics of an SMNM-based electronic system, taking a proof-of-concept demonstration of a wireless mouse application as an example. Left: Raw signals of quaternions (q_0_, q_1_, q_2_, and q_3_) obtained from the device were processed to produce representative vital signs for gesture recognition. Right: Photo showing control of the Sokoban pushing box game through hand gesture recognition (i.e., moving up, down, left, and right) [134]. Copyright 2022, Science Advances.

Song et al. [134] developed a laminated network packaging technique for miniaturized, highly integrated flexible electronic devices. They used stacked multilayer network materials (SMNMs) as a general framework for integrating and packaging inorganic stretchable electronic devices (Figure 17A). Figure 17B illustrates the integration and encapsulation of stretchable interconnects with soft network materials. The network material consisted of a periodic triangular lattice of horseshoe-shaped microstructures (PI, 50 μm thickness), with three serpentine interconnects (elements of copper) soldered to the network material by three hollow pads. The interconnects were capable of relatively free deformation under external stretching due to weak van der Waals interactions dominating the interconnect/network and network/network interfaces. Subsequently, silver posts were used to connect the holes through the circuit layers, completing the assembly of multiple circuit layers. The resulting highly integrated, miniaturized, and stretchable multilayer flexible device system (Figure 17C,D) featured ultra-high area coverage (about 110%) and a compact size (11 mm × 10 mm). Furthermore, it incorporated a compass display, somatosensory mouse control, and physiological signal monitoring (Figure 17E). The SMNM-based design strategies have broad applicability in various electronic devices, particularly those requiring high functional density, such as virtual reality devices and human-machine interfaces. Moreover, the optimization of SMNM’s three-dimensional shape for integration with biological organs, such as blood vessels and nerve conduits, showcases the promising future applications of this research and suggests potential directions for the advancement of flexible electronics.

Nowadays, research on flexible devices is flourishing, and many simple flexible products have already reached maturity and are widely used. To further expand the flexible market and enhance the functionality of flexible devices, the development of multi-layer design technology is crucial. The aforementioned examples emphasize two key aspects of the multilayer design process: effective integration of various flexible manufacturing methods and optimization of the electrical connection between layers.

For example, the combination of layer-by-layer printing and laser ablation offers a promising solution for integrating multi-layer circuit designs. This involves using laser ablation to create holes, which are then filled with liquid metals like gold and silver to establish vertical interconnections between layers. Although the multi-layer circuit design process may appear complex, it is built upon existing, mature processes. By addressing these two challenges and streamlining the design and integration of multi-layer circuits, we can undoubtedly pave the way for highly integrated, multifunctional, and reliable multi-layer flexible electronic devices.

## 4. Conclusions

In this review, we comprehensively summarize recent advances in flexible fabrication methods. These include extraction into film, self-assembly, spinning, scraping, spin coating, spray coating, deposition, print manufacturing (including screen printing, gravure printing, and inkjet printing), etching, laser, flexible electrolyte, multilayer design, and other techniques. We not only present examples of these flexible device preparation processes but also evaluate their respective strengths and weaknesses, as well as analyze methods for improvement and their prospects. Furthermore, when designing flexible products, it is essential to consider the specific characteristics of the desired product and select the most appropriate fabrication process accordingly.

1. The selection of appropriate preparation methods for flexible products is crucial and should be based on specific conditions. For the preparation of flexible films, surface uniformity and thickness are important considerations. If precision is not a concern, simple and easy operation methods such as film extraction, solution self-assembly, and spinning can be used. Additionally, simple surface treatments like scraping, spin coating, spray coating, and deposition can be employed to enhance the performance or functionality of existing films. Chemical vapor deposition, despite its high equipment costs, is widely used for treating substrate surfaces to ensure high purity of the prepared products and coat complex substrate surfaces. For the preparation of patterns with high surface complexity on flexible substrates, various processes are available. Low-resolution patterns can be achieved by combining etch-resistant materials and etching solutions with printing processes like screen printing and gravure printing. Higher precision and more complex patterns can be achieved using technologies like inkjet printing and laser processing. For example, photolithography can be used to produce nano-level precision products.

2. In addition to the preparation of thin film self-assembly and the treatment or pattern design on the film surface, the flexible preparation process and its products can be extended according to application requirements. This includes the multilayer preparation process and flexible energy storage design. While the precision and complexity of the manufacturing process for flexible energy storage devices are lower compared to other flexible electronics, the design is still challenging. Key components such as electrodes and current collectors need to be improved for flexibility. One solution is to incorporate quasi-solid electrolytes, which not only meet flexibility requirements but also prevent potential risks such as poor stability and electrolyte leakage. Furthermore, enhancing the performance of the quasi-solid electrolyte can further improve the overall performance of the flexible energy storage device. Additionally, the current functional integration of flexible electronic products is inadequate. The effective cooperation of various manufacturing processes can solve the multilayer design and manufacture of flexible devices. Ultimately, multi-layer-designed products are not only compact but also offer the integration of more functions. They possess controllable thickness, high flexibility, and durability, making them a promising direction for the future development of flexible products.

3. Although there have been numerous production processes developed for flexible electronics, many of these methods still have drawbacks and limitations. On one hand, methods such as extraction, coating, and spray plating are widely used due to their simplicity and low cost. However, these methods are only suitable for simple substrates with low requirements. If these processes are improved to meet higher standards, it will inevitably increase production processes and costs, which would negate their original advantages. Thus, striking a balance between production cost and product quality is a crucial consideration for these processes. On the other hand, processes that require special plate-making, such as gravure printing, and high-standard processes like inkjet printing, laser, and chemical vapor deposition are costly and not cost-effective. It is not realistic to expect a significant reduction in equipment costs for these processes shortly. Additionally, issues such as the difficult maintenance of inkjet printing equipment and the lack of mass production capabilities for chemical vapor deposition further increase the cost and complexity of preparation. Therefore, these challenges should be given additional attention in commercial production.

4. The development of the flexible market has had a significant impact on smart detection devices, particularly biosensors. These devices are used for a range of applications, including monitoring daily life and sports activities when attached to the human body, as well as for medical diagnosis and treatment when embedded within human tissue. To meet these diverse needs, it is essential for detection equipment to possess flexibility, stretchability, and biocompatibility. Many of the flexible device preparation methods mentioned earlier have a direct connection to biosensors and contribute to their improvement in various aspects, such as conductivity, stretchability, and biocompatibility. These methods enable the design of biosensors with enhanced performance, allowing individuals to lead healthier lives.

Over the past two decades, the flexible field has experienced significant growth and development. This review aims to provide an overview of the commonly used methods in flexible manufacturing and highlight the applications of various flexible products. By doing so, we hope to provide readers with a better understanding of the field and inspire more researchers to engage in flexible design.

## Data Availability

Not applicable.
